# Factors affecting costs for on-farm control of salmonella in Swedish dairy herds

**DOI:** 10.1186/s13028-015-0118-y

**Published:** 2015-06-06

**Authors:** Estelle C.C. Ågren, Jan Johansson, Jenny Frössling, Helene Wahlström, Ulf Emanuelson, Susanna Sternberg-Lewerin

**Affiliations:** Department of Disease Control and Epidemiology, National Veterinary Institute, SE-751 89 Uppsala, Sweden; Department for Rural Development, Swedish Board of Agriculture, SE-55182 Jönköping, Sweden; Department of Animal Environment and Health, Swedish University of Agricultural Sciences, P.O. Box 234, SE-532 23 Skara, Sweden; Department of Clinical Sciences, Swedish University of Agricultural Sciences, SE-750 07 Uppsala, Sweden; Department of Biomedical Sciences and Veterinary Public Health, Swedish University of Agricultural Sciences, SE-750 07 Uppsala, Sweden

**Keywords:** Salmonella, Costs, Control, Cattle

## Abstract

**Background:**

The Swedish control program for salmonella includes restrictions and on-farm control measures when salmonella is detected in a herd. Required control measures are subsidised by the government. This provides an opportunity to study costs for on-farm salmonella control. The aim of this study was to describe the costs for on-farm salmonella control in Swedish cattle herds and to investigate the effects of herd factors on these costs in dairy herds.

**Results:**

During the 15 years studied there had been a total of 124 restriction periods in 118 cattle herds; 89 dairy herds, 28 specialised fattening herds and three suckler herds. The average costs per herd for on-farm salmonella control was 4.60 million SEK with a median of 1.06 million SEK corresponding to approximately 490 000 and 110 000 EUR. The range was 0.01 to 41 million SEK corresponding to 1080 EUR to 4.44 million EUR per farm. The costs cover measures required in herd-specific control plans, generally measures improving herd hygiene. A mixed linear model was used to investigate associations between herd factors and costs for on-farm salmonella control in dairy herds. Herd size and length of the restriction period were both significantly associated with costs for on-farm control of salmonella with larger herds and longer periods of restrictions leading to higher costs. Serotype detected and administrative changes in the Swedish Board of Agriculture aiming at reducing costs were not associated with costs for on-farm salmonella control.

**Conclusions:**

On-farm control of salmonella in Swedish cattle herds incurred high costs but the costs also varied largely between herds. Larger herds and longer restriction periods increased the costs for on-farm control of salmonella in Swedish dairy herds. This causes concern for future costs for the Swedish salmonella control program as herd sizes are increasing.

## Background

Salmonella has long been recognised as an important food-borne zoonotic pathogen of economic significance. During the last decade increasing efforts have been made to control salmonella along the food chain within the European Union [1]. In Sweden a salmonella control program has been running since the 1960ies and it has resulted in a very low prevalence of salmonella in food of animal origin [2]. The Swedish salmonella control program comprises all serotypes throughout the chain from feed to food. Recent Swedish studies have investigated the cost-benefit of the Swedish salmonella control program and found the program as a whole to be cost- efficient [3]. However, several Swedish reports have addressed the increasing costs for the part of the national control program that concerns the on-farm control of salmonella [4, 5].

When salmonella is detected in a Swedish herd, the holding is put under restrictions and a control plan is put in place. The government partly subsidises the farmers for measures that are required during the restriction period. The costs for on-farm control of salmonella in cattle herds is the second most costly part of the Swedish salmonella control program despite only a small number of positive herds detected each year. Only the feed control incurs higher costs [3]. Cattle are the species where most salmonella herds are detected and consequently also the species where most money is spent on control measures and therefore the focus of this study [2].

Economic losses caused by salmonella infections in cattle herds have been well investigated by others [6]. However, to the authors’ knowledge no detailed analyses of documented costs for on-farm control of salmonella have been published. The Swedish system provides an opportunity to estimate these costs from subsidises paid to the farmers.

The aim of this study was to describe the costs for on-farm salmonella control in Swedish cattle herds and to investigate the effects of herd factors on these costs.

This paper includes a description of all Swedish cattle herds that have been under restrictions due to salmonella during the last 15 years and a regression analysis of the associations between herd factors and costs for on-farm control of salmonella in dairy herds.

## Methods

### The Swedish salmonella control program

Within the Swedish program, infected cattle herds are detected by faecal sampling on clinical suspicion, sampling of dead calves at necropsy, sampling of lymph nodes at slaughter and through tracings from different sources such as contaminated feed, infected herds, contaminated meat and infected humans. A culture positive on-farm sample results in the herd being put under restrictions and a herd-specific control plan is drawn [7, 8]. The plan includes general measures for improving herd hygiene, but the specific measures required vary between herds. Commonly required measures are listed in Table 1. The Swedish Board of Agriculture (SBA) determines the herd-specific control plan, but this can also be delegated to the county veterinarian at the County Administrative Board.

**Table 1:** Commonly required measures in cattle herds with restrictions due to detection of salmonella

Improved stable hygiene *i.e.* adequate cleaning and bedding materials
Hygiene barriers between different groups of animals *e.g.* change of boots and separate tools
Separate tools and vehicles for handling feed and manure
Improved feed hygiene *i.e.* a clean feed chain including a clean feeding table
Improved water hygiene including clean drinking cups
Having a control program for pests in place
Avoiding overcrowding of animals in stables
Good management routines around calvings including early removal of calves from their dams
Good management and feeding routines for new born calves
Keeping areas around stables clean
Keeping fences around pastures intact
Hygienisation of manure before surface spread
Thorough cleaning of stables at the end of restrictions including replacement of fittings that cannot be properly cleaned

The farmer can claim financial compensation for costs caused by measures stated in the herd specific control plan. Examples of costs that will be compensated are costs for increased working hours, culled animals in cases of overcrowding, production losses due to decreased number of animals, costs for thorough cleaning of stables and replacement of materials that cannot be properly cleaned as well as costs for hygienisation of manure and compensation for loss of its value as fertilizer. The financial compensations paid by the SBA cover either 50 % or 70 % of the eligible claimed costs [9]. The higher level of compensation is paid to farmers affiliated to a voluntary salmonella program focusing on preventive measures. To be eligible for compensations herds may not have bought more than 150 animals from more than five herds within the last year. Compensations have to be claimed within six months after removal of restrictions. Restrictions are removed when two consecutive rounds of faecal samples from all animals in the herd are negative on bacteriological culture. In herds where separate epidemiological units exist, restrictions may be lifted on parts of the herd.

In 2009 changes in the administration in the SBA were initiated. The purpose of these changes were to reduce costs. These changes included a more restrictive approach to financial compensations, less focus on major cleaning and more focus on daily hygiene routines in the herds.

### Data

All cattle herds put under restrictions due to positive on-farm samples during the last 15 years (1999–2013) were included in the study.

Data on herd identities, geographic location (county), type of production (dairy herd/specialised fattening unit/suckler herd), salmonella serotype detected and dates when restrictions were initiated and removed were retrieved from records kept at the National Veterinary Institute (SVA). In most of the herds that were put under restrictions only one serotype was detected, isolated findings of a second serotype was not included. If restrictions had been partially removed, the date when restrictions on all animals had been removed was used.

Data on herd size at the time restrictions were initiated, financial compensations paid by the SBA and the SBA level of compensation were compiled from documentation at the SBA, available from 1999 and onwards. Only herds where the deadline for claiming financial compensation had been passed were included.

### Data editing

When the number of cows, but not the total number of animals was known, the herd size was estimated by multiplying the number of cows by two according to documented herd structure in Swedish dairy herds [10].

In order to get comparable costs between herds that had received 50 % and 70 % compensations, all compensations paid by the SBA were recalculated as if all herds had received 100 % compensation, *i.e.* dividing the actual costs by 0.5 and 0.7 respectively. Thereafter, in order to compensate for inflation, the costs for each herd were recalculated to a value corresponding to year 2013, using the official Swedish consumer price index [11] at the midpoint of the restriction period.

In the regression analysis only dairy herds were included, because conditions in non-dairy herds are different in a way considered likely to affect costs. Only dairy herds that had received financial compensations were included. The number of non-dairy herds that had received financial compensations was too small for a separate analysis. Three dairy herds had repeated periods of restrictions, in these herds one randomly selected restriction period was included in the statistical analyses.

### Statistical analysis

**Fig. 1:**
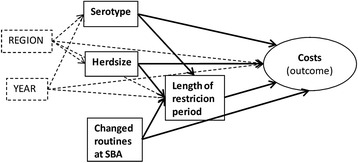
Causal diagram showing all variables analysed for associations with costs for on-farm control of salmonella in dairy herds. Region and year were included as random effects

A causal diagram (Fig. 1) was drawn to guide the statistical analysis. All variables with available information have been included in the diagram. All these variables were considered to be of interest to evaluate and were therefore included in the model.

A mixed linear model was used to evaluate potential associations between herd factors and the costs during the restriction period in dairy herds. The recalculated costs was the outcome variable and the fixed explanatory variables included in the model were herd size, length of the restriction period, serotype detected in the herd and changes in the administration at the SBA. Herd size (number of animals) and restriction period (number of days) were included as continuous variables. Serotype detected in the herd was included as a categorical variable with four levels, *S.* Dublin, *S.* Typhimurium (including monophasic *S.* Typhimurium), *S.* Reading and “other serotypes”. “Other serotypes” included seven serotypes with one or two observations each. A categorical variable was created for herds being put under restrictions before and after changes in administration at the SBA in January 2009 as a way to evaluate if these changes had resulted in lower costs as intended. County and year for initiation of restrictions were included as random effects to adjust for possible differences but not estimate their effects.

All continuous variables (costs, herd size and restriction period) were log-transformed in order to achieve normally distributed and homoscedastic residuals and to improve model fit. Confounding was tested according to Dohoo *et al.* [12]. In brief, potential confounders were examined for confounding by looking at the change in parameter estimates with and without controlling for the potential confounder. Interactions between all fixed effects were tested but none were significant or improved the model fit significantly as evaluated by the Akaike Information Criterion (AIC), so no interaction terms were included in the model. Parameter estimates and their standard errors were checked for signs of multicollinearities in the model, but no such indications were found. Residuals were checked for normality and homoscedasticity but no deviations from assumptions were detected. Proportion explained variance was calculated according to Snijders and Bosker [13] by comparing the proportion unexplained variance in the full model with the proportion unexplained variance in a model with only random effects.

Predictions were made to illustrate the effect of herd size and length of restriction period on costs. Predictions were obtained as linear combinations of the parameter estimations from the model evaluated at herd sizes of 50–1150 animals with 100 animal intervals and length of restriction periods at 50–1450 days with 150 day intervals. Predictions were made with serotype set to Dublin, county set to Kalmar (H), year set to 2013 and changes in administration at the SBA set to after changes were performed in 2009.

All statistical analyses were performed in R using the R package “lmerTest” for the regression analysis [14, 15]

## Results

### Descriptive statistics of all cattle herds in the study

**Fig. 2:**
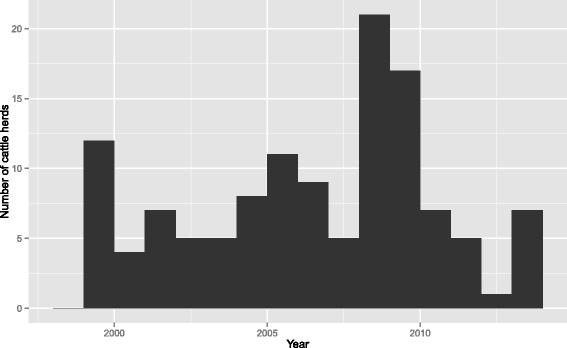
Number of salmonella infected cattle herds detected per year in Sweden 1999–2013

**Fig. 3:**
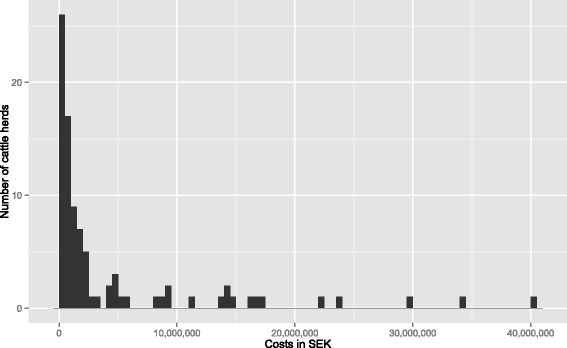
Distribution of costs for subsidised on-farm control measures. All cattle herds with restrictions due to salmonella in Sweden 1999–2013 have been included

During the 15 years 1999–2013 there has been a total of 124 restriction periods in 118 herds with positive on-farm samples. In Fig. 2 the number of herds detected each year is shown. The average cost for all herds was 4.60 million SEK with a median of 1.06 million SEK corresponding to approximately 490 000 and 110 000 EUR [16]. The large difference in average and median value is due to a small number of herds with very large costs, the range of costs being 0.01 to 41 million SEK corresponding to 1080 EUR to 4.44 million EUR (Fig. 3). In Table 2 the number of herds, restriction periods as well as herd sizes, length of restriction periods and costs for the different types of production are summarised. In Table 3 more detailed results for dairy herds is shown including number of herds for each serotype and county.

**Table 2:** Characteristics of Swedish cattle herds with restrictions due to detection of salmonella 1999–2013

Production	No of restriction periods (No of herds)	No of restriction periods with financial compensations	Herd size (no of animals) median(min max)	Restriction period (days) median(min max)	Costs (mSEK) median (min max)
Dairy herds	92 (87)	80	237 (35–1008)	256 (49–1495)	1.06 (0.01–40.10)
Specialised fattening units	29 (28)	8	114 (36–632)	253 (106–667)	0.74 (0.05–4.31)
Suckler herds	3 (3)	2	419 (151–687)	423 (88–758)	8.47 (0.11–16.80)
Total	124 (118)	90	231 (35–1008)	256 (49–1495)	1.06 (0.01–40.10)

**Table 3:** Characteristics of Swedish dairy herds with restrictions due to detection of salmonella 1999-2013

Serotype	County (no of herds)	No of restriction periods	Herd size (no of animals) median(min max)	Restriction period (days) median(min max)	Costs (mSEK) median(min max)
Dublin	C (1)	1	118	226	0.32
	D (6)	6	101 (86–278)	187 (87–1136)	1.04 (0.04–4.94)
	E (3)	5	612 (204–735)	567 (245–939)	9.26 (1.64–17.50)
	G (3)	3	191 (117–637)	405 (138–467)	2.18 (0.58–9.34)
	H (24)	25	230 (89–474)	221 (49–1015)	0.65 (0.01–2.43)
	O (2)	2	336 (203–470)	325 (246–405)	7.52 (0.78–14.30)
Typhimurium	C (1)	1	49	176	0.75
	E (1)	3	600 (600–650)	804 (434–1184)	16.50 (14.10–22.00)
	G (2)	2	299 (106–493)	193 (142–244)	0.70 (0.50–0.90)
	H (5)	5	225 (128–1008)	281 (90–733)	2.27 (0.23–5.81)
	K (1)	1	240	1121	4.52
	M (4)	4	363 (41–605)	282 (73–500)	1.45 (0.13–29.80)
	N (1)	1	265	298	1.58
	O (7)	7	230 (89–750)	295 (72–645)	1.05 (0.10–23.90)
Reading	M (5)	5	361 (300–603)	1052 (720–1495)	8.85 (3.28–40.10)
Other serotypes	AB (1)	1	190	161	0.80
	F (1)	1	90	115	0.53
	M (4)	4	231 (127–352)	272 (70–347)	1.69 (0.12–11.10)
	O (2)	2	301 (103–500)	504 (264–744)	17.5 (0.86–34.20)
	X (1)	1	35	55	0.01

Four herds, three dairy herds and one specialized fattening unit, had repeated periods of restrictions. One of the dairy herds had four separate periods of restrictions, the other three herds had two restriction periods each.

Compensations were not paid for twelve of the 92 restriction periods in dairy herds (13 %) and for 21 of the 29 restriction periods in specialized fattening units (72 %). Based on experience the most common explanation for this would be that the specialized fattening units were not eligible for compensations due to purchase of too many animals. For dairy herds a likely reason would be a low grade of infection in the herd and consequently only very limited control measures undertaken.

### Regression analysis of costs in dairy herds

In total 75 dairy herds were included in the statistical analysis. Serotype was clustered on county, more than half the herds with *S*. Dublin were detected in the county of Kalmar (H) and all herds with *S.* Reading were detected in the county of Skåne (M) (Table 3). Herds with *S.* Reading were detected only during the years 2007 to 2011 during an outbreak in the county of Skåne (M). *Salmonella* Dublin, *S*. Typhimurium and “other serotypes” were detected throughout the 15 years studied.

**Fig. 4:**
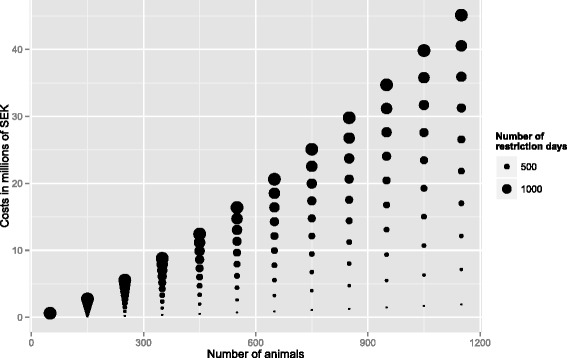
Model predictions. Model predictions illustrating the effect of herd size (x-axis) and length of restriction period (size of dots) on costs for controlling salmonella (y-axis) in dairy herds. Predictions are from a mixed linear regression model and are performed with serotype = Dublin, county = county of Kalmar (H), administrative change at the SBA = 2009–2013 and year = 2013

Results from the model are shown in Table 4. Neither serotype nor changes at the SBA were significantly associated with costs but were kept in the final model as they were factors of primary interest and also were considered a priori to be potential confounders. The fixed part of the model explained 69 % of the variance in the data. Both herd size and length of the restriction period were significantly associated with costs with larger herds and longer restriction periods associated with higher costs. Correcting for herd size changed the estimate for the length of the restriction period with 38 % indicating that herd size was associated not only with costs, but also with length of the restriction period. Serotype was not significantly associated with costs and correcting for serotype changed the estimate for the length of the restriction period with only 7 % indicating that serotype was not associated with costs directly and did not modify the effect of the length of the restriction period. In Fig. 4 model predictions for costs in herds with different sizes and different lengths of restriction period is shown.

**Table 4:** Results from regression analysis of factors affecting costs for salmonella control in Swedish dairy herds

*Random effects:*					
Group	Variance	Std Dev			
Year	0.28	0.53			
County	0.10	0.31			
Residual	0.88	0.93			
*Fixed effects:*					
Variable	Category	Estimate	Std Error	t-value	*p*-value
Intercept		0.85	1.16	0.73	0.46
Herd size		1.37	0.20	6.71	<0.001
Restriction period		0.95	0.19	5.02	<0.001
Serotype	Dublin	Ref level	-		-
	Typhimurium	0.38	0.29	1.27	0.21
	Reading	0.18	0.61	0.29	0.77
	Other^a^	0.80	0.43	1.86	0.07
Administrative change at SBA	1999–2008	Ref level	-		-
	2009–2013	0.24	0.45	0.55	0.59

A mixed linear model was used. County and year restrictions were initiated were included as random effects. All numerical variables were log-transformed in the model. All factors of interest where data were available were included and kept in the model, although some did not show a significant association with the outcome

^a^“Other” included seven serotypes with one or two observations each

## Discussion

### Costs

The results from this study provide opportunities for a rough comparison of costs for on-farm control of salmonella versus production losses reported by others. Nielsen *et al.* reported economic losses caused by *S.* Dublin infection during a 10-year period in a dairy herd with 200 cows adding up to 27 600 EUR in a herd with good hygiene and 415 800 EUR in a herd with poor hygiene [6]. Using predictions from the model in this study, a herd with 400 animals (200 cows) infected with *S.* Dublin would have costs for controlling salmonella ranging from 170 000 to 510 000 EUR with the lower costs for a shorter restriction period of 200 days and the higher costs for a longer restriction period of 650 days, a timespan that covers the length of the restriction period for most herds being put under restrictions. These predicted costs are largely within the range of previously reported production losses.

### Herd size

Herd size was the variable with the largest effect on costs (Table 4). Increasing herd size was associated with higher costs and this effect was more pronounced for larger herd sizes (Fig. 4). The effect of herd size was still significant when a regression analysis was performed with the outcome changed to cost per animal indicating that it is not only the total cost that increases, but also the cost per animal. The results also showed that herd size had an indirect effect on costs by prolonging the restriction period. At present the herd sizes of Swedish cattle herds are increasing [17]. Therefore this finding causes concern as it implies increasing costs for the Swedish control program in the future if the present on-farm strategies are preserved.

### Restriction period

A longer restriction period was associated with higher costs, which was quite as expected. The effect was significant also after correcting for herd size. The length of the restriction period probably reflects several other important factors. Boqvist *et al.* showed that herd size, abundance of birds and rodents and several farm sites were associated with a longer restriction period [18]. Other influential factors could be herd management and hygiene, stalling type, type of milking system and within-herd prevalence at detection. Moreover, the individual commitment of the farmer, appointed herd veterinarian, county veterinarian or veterinarian at the SBA could have impact on the length of the restriction period. This was reported in a Swedish project were interviews with people involved in on-farm salmonella control were performed [19]. The driving force of involved people was considered an important factor for the length of the restriction period by many of the interviewed. Furthermore, a system where the on-farm control measures are governed by the authorities instead of the farmers’ own initiatives might result in reduced motivation and longer restriction periods [20, 21].

### Serotype

Serotype had no significant effect on costs. In order to evaluate if the costs were different for herds with *S.* Dublin compared to all other serotypes the model was also run with serotypes divided in only two categories. Serotype still had no significant effect on costs. One explanation for the lack of significant differences could be a small number of herds infected with some of the serotypes (Table 3) which reduces the likelihood of finding significant differences. However, when the analysis was performed with only two categories of serotype the groups were much larger, and the difference was still not significant. This study evaluates costs for required measures within the Swedish control program and these measures are similar in herds with different serotypes. Therefore the costs may not be expected to be very different for different serotypes. The cattle adapted serotype *S.* Dublin may be expected to persist longer in a cattle herd than other serotypes [22] and therefore cause higher costs. However, this study did not show any signs of higher costs due to longer restriction periods in herds with *S.* Dublin infections either. An explanation for this might be a lower diagnostic sensitivity for *S.* Dublin versus other serotypes [23–25] resulting in premature lifting of restrictions in herds with *S.* Dublin. Serological testing in herds where restrictions have been lifted suggests that this may be the case.

Differences in herd size in herds infected with different serotypes are not considered to contribute to the lack of significant differences in costs between serotypes, as herd size was corrected for in the analysis.

As serotype was clustered on county and county was included as a random factor there was concern that the effect of serotype might be underestimated. However, the proportion of variance explained by county was reduced from 30 % to 8 % when the fixed part of the model was added. This indicates that a large part of the variation on county level is explained by the fixed variables. The effect of serotype is therefore, not considered likely to be underestimated.

### Change of routines at the SBA

The change of administrative routines at the SBA in 2009 showed no significant effect on costs in this study. It shows that despite intense efforts by the SBA it was difficult to achieve changes in the established system that would reduce the costs for on-farm control of salmonella. In this study, only subsidises paid to the farmers were evaluated, other possible benefits *e.g.*in the administration at the SBA was not evaluated.

### Other aspects to consider

Removal of restrictions from a herd is based on negative culture results. Due to poor diagnostic sensitivity of culture on individual animals [24] culture is only used on herd basis in the Swedish control program as this improves the sensitivity. Also, sampling is repeated twice before restrictions are lifted. Despite this, it is possible that some herds, particularly with *S.* Dublin, may not be free from salmonella when restrictions were lifted. We therefore use the term on-farm control of salmonella, even if the long term aim is eradication from the herd.

The costs shown in this study might be higher than necessary for on-farm control of salmonella. When designing a herd-specific control plan it is difficult for the appointed veterinarian to decide which measures are necessary and which are not, and this may lead to application of the precautionary principle. Consequently excessive measures might have been included in the herd-specific control plans. Moreover, during the 1990s companies handling cleaning and disinfection of stables entered the market and with time most farmers engaged these companies to perform the cleaning and disinfection required in the herd-specific control plan. These companies often performed meticulous cleaning procedures excessive to what is necessary for on-farm control of salmonella in cattle herds. This could also have caused higher costs than necessary.

Data was only available on a few factors when in reality there is a large number of factors that will affect the costs for control. Examples of such factors are within-herd prevalence at detection, herd hygiene and type of stalling. However, many of these factors are likely to be reflected in the length of the restriction period which was included in the regression analysis.

## Conclusions

Mandatory on-farm control of salmonella in Swedish cattle herds incurs high costs but also varies largely between herds. Larger herds and longer restriction periods increased the costs for on-farm control of salmonella in Swedish dairy herds. This causes concern for increasing costs for the Swedish salmonella control program in the future as herd sizes are increasing.

## Abbreviations

SBA: Swedish Board of Agriculture
SVA: Swedish National Veterinary Institute
